# Volatility impacts on the European banking sector: GFC and COVID-19

**DOI:** 10.1007/s10479-022-04523-8

**Published:** 2022-02-18

**Authors:** Jonathan A. Batten, Tonmoy Choudhury, Harald Kinateder, Niklas F. Wagner

**Affiliations:** 1 RMIT University College of Business, Melbourne, VIC Australia; 2 IRC of Finance and Digital Economy, King Fahd University of Petroleum & Minerals, Dhahran, Kingdom of Saudi Arabia; 3 School of Business, Economics and Information Systems, University of Passau, Passau, Germany

**Keywords:** COVID-19, Swine Flu (H1N1), Zika virus, GFC, DCC-GARCH, Europe, Global systemically important banks, Implied volatility, C22, G32

## Abstract

This paper analyses the volatility transmission between European Global Systemically Important Banks (GSIBs) and implied stock market volatility. A Dynamic Conditional Correlation Generalized Autoregressive Conditional Heteroskedasticity model is applied to determine the dynamic correlation between returns of Europe’s GSIBs and the world’s most prominent measure of market “fear”, the CBOE Volatility Index (VIX). The results identify a higher negative co-relationship between the VIX and GSIB returns during the COVID-19 period compared with the Global Financial Crisis (GFC), with one-day lagged changes in the VIX negatively Granger-causing bank returns. The asymmetric impact of changes in implied volatility is examined by quantile regressions, with the findings showing that in the lower quartile–where extreme negative bank returns are present–jumps in the VIX are highly significant. This effect is more pronounced during COVID-19 than during the GFC. Additional robustness analysis shows that these findings are consistent during the periods of the Swine Flu and Zika virus epidemics.

## Introduction

The European banking sector is one of the world’s largest and is an integral part of the global financial system, while also incorporating some of the world’s largest economies.^1^ Previous empirical research has highlighted the importance of risk management given potential contagion effects with banks both within and outside Europe (Gabrieli & Salakhova, 2019; Teply & Klinger, 2019). In fact, the OECD (2021) has noted that as a consequence of the recent COVID-19 pandemic, there has been an increase in household and corporate default that has had a direct impact on bank asset quality and added to regulatory concerns over domestic and international financial system stability.

In this paper, we investigate the volatility impacts on the European banking sector, with specific attention to the COVID-19 crisis period. We add to recent work on volatility transmission within the banking industry more generally, as well as recent work on financial market impacts during the COVID-19 crisis (Claeys, 2020). The recent crisis has changed the nature and scope of the financial industry due to policy interventions aimed at reducing tightening of funding conditions (Demirguc-Kunt et al., 2020). A unique feature of this study is the identification of the time-varying correlation between implied stock market volatility and the largest European banks, defined by banking regulators as those that are Global Systemically Important Banks (GSIBs). Furthermore, the study compares patterns in transmission during the Global Financial Crisis between 2007 and 2009, and the COVID-19 period.

The results show that there was a higher negative co-relationship between VIX and GSIB returns during the COVID-19 period compared with the Global Financial Crisis (GFC). Thus, the impact of COVID-19, so far, has been significantly different from those experienced during the Global Financial Crisis (GFC) (e.g. Dinçer et al., 2019; Tan et al., 2020).

This study uses the Chicago Board Options Exchange's (CBOE) Volatility Index (VIX) as a measure of the United States (US) stock market’s expectation of volatility. This index is commonly known as the “fear index” or “fear gauge” (Ding et al., 2021; Simon & Wiggins III, 2001; Whaley, 2000, 2009), and has been used to study volatility transmission during previous crisis periods (Cheuathonghua et al., 2019; Rodriguez-Nieto & Mollick, 2020), as well as to measure the transmission of volatility between financial entities (Kang et al., 2019; Pan et al., 2019). One key contribution of this paper is that we establish how changes in correlation patterns between European banks and the VIX occurred during the GFC and COVID-19 periods.

In addition, we determine if lagged movements in the VIX, Granger-cause bank returns. For this purpose, we apply Engle’s (2002) Dynamic Conditional Correlation (DCC), Generalized Autoregressive Conditional Heteroskedasticity (GARCH) model. Since constant correlations are not supported empirically (e.g. Lin et al., 2014), this improved approach allows better estimation and more accurate reporting of the dynamic correlations. In a second step, we use a quantile regression model to provide a more detailed impression of how bank returns react to changes in implied volatility. During the GFC and COVID-19 periods, there were significant increases in market “fear” among investors.^2^ Therefore, it is important to determine if market reactions are asymmetric, with market participants reacting more to negative news compared with positive news.

We analyze the daily returns of the ten most prominent European GSIBs for the period from January 1, 2002, to May 21, 2021. To avoid a bias due to stationarity and the presence of unit-roots, we focus on bank returns and relative VIX changes, i.e. returns. Our results demonstrate that bank returns are significantly negatively related to lagged VIX returns. This is consistent with lagged movements in the VIX, Granger-causing bank returns. Changes in the correlation patterns, between bank and VIX returns, during both crises are similar, although there is a higher negative correlation for some banks during the COVID-19 period. The outcomes of the quantile regressions show that jumps in contemporaneous VIX returns are significantly negatively related to bank returns at the 25% quantile. We also document asymmetry between jumps and declines in the VIX, although this asymmetry is more pronounced during COVID-19 than during the GFC. As COVID-19 was not the only epidemic that has appeared in the last decades, we also determine if the Swine Flu (H1N1) and the Zika virus also show similar effects as documented in our COVID-19 sample. For both viruses, we find a significant negative relation between bank returns and VIX jumps in the 25% quantile. However, the asymmetry between VIX jumps and declines is less pronounced for Swine flu, whereas the results in the Zika sample are like those of COVID-19.

The paper is organized as follows: the next section briefly reviews the relevant literature followed by the methodology and preliminary analysis. Section 5 contains the results of the empirical analysis. Finally, Sect. 6 concludes.

## Literature review

The volatility transmission literature is a critical component of the modern-day risk management literature (Aloui et al., 2011; Daly et al., 2019; Sensoy et al., 2019). Before the GFC, regulatory attention was directed towards improving the adequacy of risk management techniques at the individual bank level, such as through Value-at-Risk (VaR) forecasting. However, in the aftermath of the GFC there has been an increased interest in modeling systemic risk, due to higher interbank connectedness. Thus, the default of a GSIB would not remain an independent event. As a result, several papers determine the contagion or spillover effects of systemic risk in the banking sector (Ahnert & Georg, 2018; Cai et al., 2018; Gaies et al., 2019; Löffler & Raupach, 2018; Pagratis et al., 2017). A feature of this literature has been the development of special risk measures for modeling the contribution to systemic risk of a bank, through such measures as conditional VaR (Adrian & Brunnermeier, 2011) or SRISK (Brownlees & Engle, 2017).

Volatility transmission during the COVID-19 crisis is not only relevant for risk management but also for asset managers, who must establish if there are important differences to past crises (Bhattacharjee et al., 2020; Davis, 2020; Goutte et al., 2020). In this respect, a number of recent studies have investigated the relationship between the VIX and European financial markets (e.g. Cheuathonghua et al., 2019; Tissaoui & Zaghdoudi 2021), given that the VIX has become the dominant measure of risk volatility in the financial world (Bardgett et al., 2019; Wang, 2019). However, very few studies investigate the spillover between the VIX and the European banking sector. Recently, Shahzad et al. (2020) examines the connectedness between Credit Default Swap (CDS)-VIX pairwise assets and eleven US stock market sectors, including the banking sector, while Mensi et al. (2019) examine the impact of the VIX on US financial credit markets. Both studies highlight the relevance of the VIX as an impact on credit risk in the financial sector. An alternate measure of contagion to the VIX is the VSTOXX volatility^3^ (Pancotto et al., 2019; Torre-Torres et al., 2021). In this study, we prefer to use the VIX as it is a better indicator of global risk and allows comparison with other academic studies. Importantly, given its wide acceptance as a global fear index, jumps in VIX are more important to investors than jumps in VSTOXX (Aragon et al., 2020). Note that recent work has also used the VIX when examining the impacts of the GFC and COVID-19 with respect to safe-haven asset allocation (Kinateder et al., 2021).

Overall, the VIX plays a major role in risk management, especially for large international banks whose asset portfolios are more likely to be affected by international price shocks, as occurred during COVID-19 (Adrangi et al., 2021; Jeris & Nath, 2021). The current financial and banking market turmoil fueled by COVID-19 has added to investor concerns over its duration and broader market impact, such as potential distress and bankruptcy (e.g. Djalilov & Ülkü, 2021; Glossner et al., 2020). Importantly, Wang (2019) shows that VIX is a better predictor for future volatility during COVID-19 than other measures, such as the commonly used Economic Policy Uncertainty (EPU), for studies in the contemporary banking sector (Cerutti et al., 2017; Huang et al., 2021).

When working with VIX, or other implied volatility indices, another relevant point is potential volatility asymmetry. Aboura and Wagner’s (2016) analysis of daily VIX changes, finds evidence for an extreme asymmetric volatility effect, which is significant during periods of market stress. Fousekis (2020) also documents an asymmetric relationship between implied volatility indices (e.g. VIX and VSTOXX) and stock markets (i.e. negative returns are associated with higher implied volatility than positive ones). These findings are in line with other studies in other asset classes such as commodities (Yip et al., 2020) or cryptocurrencies (Gemici & Polat, 2021) and summarized in the review of key studies on implied volatility undertaken by Fassas and Siriopoulos (2021).

## Methodology

In this section, we present the methodology used in this paper. The methods employed can be divided into two sections–the primary DCC-GARCH model, which is used to determine the conditional correlation between banks’ stock returns and VIX as well as to study whether VIX returns Granger-cause bank stock returns. The GARCH model is the preferred method of correlation or contagion analysis (Abid et al., 2020; Akhtaruzzaman et al., 2020; Arouri et al., 2011). Secondly, we analyze the asymmetric impact of changes in implied volatility in bank returns using a quantile regression approach.

### DCC-GARCH model

We model the bivariate (2 × 1) vector ${\varvec{R}}_{t} = \left( {R_{s,t} ,R_{VIX,t} } \right)^{\prime}$ of conditional stock returns of bank *s*, $R_{s,t}$, and VIX returns, $R_{VIX,t}$, by a DCC-AR(4)-GARCH(1,3) model in the spirit of Engle (2002):

1 $$ {\varvec{R}}_{t} = {\varvec{\mu}}_{t} + {\varvec{H}}_{t}^{0.5} {\varvec{z}}_{t} $$

where ${\varvec{z}}_{t}$ is a (2 × 1) vector of i.i.d. innovations which is assumed to be bivariate normal, i.e. ${\varvec{z}}_{t} \sim N\left( {0,{\varvec{H}}_{t} } \right)$, see Engle (2002). The conditional covariance matrix ***H***_***t***_ can be decomposed into a (2 × 2) diagonal matrix ${\varvec{D}}_{t}$, whose elements consist of conditional standard deviations $h_{i,t}^{0.5}$ with $i \in \left\{ {s,VIX} \right\}$, and a conditional correlation matrix ${\varvec{C}}_{t}$:

2 $$ {\varvec{H}}_{t} = {\varvec{D}}_{t} {\varvec{C}}_{t} {\varvec{D}}_{t} $$

We assume that the conditional mean $\mu_{i,t} = E(R_{i,t} |{\mathcal{F}}_{t - 1} )$ is expressed as an autoregressive process of order four. Then the univariate conditional mean equation is

3 $$ R_{i,t} = \mu_{i} + \mathop \sum \limits_{j = 1}^{4} \rho_{j} R_{i,t - j} + \mathop \sum \limits_{p = 1}^{4} \lambda_{p} R_{VIX,t - p} + e_{i,t} $$

where $e_{i,t} = h_{i,t}^{0.5} z_{t}$ denotes the unstandardized innovations with mean zero and conditional variance $h_{i,t}$. We use an AR term $\mathop \sum \nolimits_{j = 1}^{4} \rho_{j} R_{i,t - j}$ up to order four to account for possible serial correlation in bank returns. The coefficient $\lambda_{1}$ is used to analyze potential Granger causality between the lagged VIX return in time *t*-1 and the stock return of bank *s* in time *t*.

We use the GARCH approach of Bollerslev (1986) to estimate the univariate conditional variances of stock and VIX returns $h_{i,t} = Var(R_{i,t} |{\mathcal{F}}_{t - 1} )$:

4 $$ h_{i,t} = \omega_{i,0} + \omega_{i,1} e_{i,t - 1}^{2} + \mathop \sum \limits_{j = 1}^{3} \omega_{i,1 + j} h_{i,t - j} $$

where all $\omega$ should be greater than zero. Our conditional correlation matrix can be stated as Eq. (5) using ${\varvec{Q}}_{t} \user2{ }$(2 × 2) covariance matrix.

5 $$ {\varvec{C}}_{t} = \left( {diag{\varvec{Q}}_{t} } \right)^{ - 0.5} {\varvec{Q}}_{t} \left( {diag{\varvec{Q}}_{t} } \right)^{ - 0.5} $$

As stated by Batten et al. (2021), if we specify ${\varvec{u}}_{t - 1}$ as a (2 × 1) vector of standardized innovations with unconditional correlation matrix $\overline{\user2{C}}$, and $u_{i,t} = \frac{{e_{i,t} }}{{\sqrt {h_{i,t} } }}$, we can derive the matrix ${\varvec{Q}}_{t}$ as

6 $$ {\varvec{Q}}_{t} = \left( {1 - a - b} \right)\overline{\user2{C}} + a{\varvec{u}}_{t - 1} \user2{u^{\prime}}_{t - 1} + b{\varvec{Q}}_{t - 1} $$

The value of the positive scalers *a* and *b* is limited to $a + b < 1$. We can attain the conditional correlations from the elements of the matrix ${\varvec{Q}}_{t}$ using Eq. (7), where $q_{s,VIX,t}$ is the conditional covariance $q_{s,VIX,t} = Cov(R_{s,t} ,R_{VIX,t} |{\mathcal{F}}_{t - 1} )$ between bank *s* and VIX and $q_{s,s,t} = Var(R_{s,t} |{\mathcal{F}}_{t - 1} )$ and $q_{VIX,VIX,t} = Var(R_{VIX,t} |{\mathcal{F}}_{t - 1} )$ represent the conditional variances of bank *s* and VIX return, respectively.

7 $$ \rho_{s,VIX,t} = q_{s,VIX,t} /\left( {q_{s,s,t} q_{VIX,VIX,t} } \right)^{0.5} $$

Based on these estimates, we can compute the DCC-AR(4)-GARCH(1,3) model for our baseline result.

### Asymmetric impact of changes in implied volatility on bank returns

In the final stage, we analyze the asymmetric impact of changes in implied volatility on bank returns. To analyze the asymmetric response of bank returns to relative changes in VIX, we define two new variables: relative positive and relative negative changes in VIX as $VIX_{U,t} = {\text{max}}(R_{VIX,t} ,0)$ and $VIX_{D,t} = {\text{min}}(R_{VIX,t} ,0)$, where $R_{VIX,t}$ denotes the daily VIX return. In an ordinary OLS regression, the mean of the dependent variable is regressed on a set of independent variables. This is needed if the distribution is symmetric and one does not expect different market reactions to have extremely negative and positive returns, respectively. If there is increased fear among investors of price declines during a crisis period, there will be a more pronounced reaction to negative news compared to positive news. As a result, we use a quantile regression model to establish how bank returns react to changes in implied volatility. The baseline regression model is specified as follows:

8 $$ R_{s,t} = \beta_{0} + \beta_{1} VIX_{U,t} + \beta_{2} VIX_{D,t} + \varepsilon_{s,t} $$

where $R_{s,t}$ is the return of bank *s* and $\varepsilon_{s,t}$ denote the residuals. As we estimate Eq. (8) as quantile regression, the estimated quantile of the dependent variable’s distribution conditional on the values of the independent variables is

9 $$ Q_{\tau } [R_{s,t} |VIX_{U,t} ,VIX_{D,t} ] = \hat{\beta }_{0} + \hat{\beta }_{1} VIX_{U,t} + \hat{\beta }_{2} VIX_{D,t} $$

where $Q_{\tau } [R_{s,t} |VIX_{U,t} ,VIX_{D,t} ]$ is the predicted $\tau$-th quantile.

Since $\tau \in \left( {0,1} \right)$, we can perform a rich analysis of different parts of the distribution. Due to non-symmetric outliers, ordinary OLS regression can be biased, therefore the median is often a better choice than the mean. As a result, we focus on the median (i.e. 50% quantile) as well as the 25% and 75% quantile. The last two quantiles are used to analyze bank returns that are significantly different from zero. Since not only positive and negative jumps in VIX can show different results, but also extreme negative and positive bank returns, we also analyze the 25% and 75% quantiles.

## Data and preliminary analysis

We collect daily closing prices $P_{i, t}$ from DataStream consisting of the ten most prominent European GSIBs for the period from January 1, 2002, to May 21, 2021.^4^ The usage of daily data allows us the modeling of conditional correlations in both crisis periods. The GSIBs investigated comprise the CREDIT SUISSE GROUP (CSG), UBS (UBS), BANCO SANTANDER (BST), ING GROUP (ING), UNICREDIT (UNI), DEUTSCHE BANK (DTS), BNP PARIBAS (BNP), CREDIT AGRICOLE (CRA), SOCIETE GENERALE (SGR) and NATIXIS (NAT). We have chosen NAT as the largest listed substitute of the Group BPCE. Moreover, we have chosen only banks from continental Europe and ignored British banks to avoid any biases due to Brexit.

Table 1 presents key descriptive statistics for the full sample period and two separate subsamples of the Global Financial Crisis (GFC) and COVID-19. We report statistics for daily continuously compounded returns of bank stocks as well as the VIX, which are computed as $R_{i,t} = {\text{ln}}(P_{i,t} ) - {\text{ln}}(P_{i,t - 1} )$. The GFC sample is from August 1, 2007, to December 2, 2008, which includes the collapse of Lehman Brothers in September 2008, while the COVID-19 sample consists of daily data from January 1, 2020, to May 5, 2021.

**Table 1:** Descriptive Statistics

	Credit suisse group	Ubs	Banco santander	Ing group	Unicredit	Deutsche bank	Bnp paribas	Credit agricole	Societe generale	Natixis	Vix
*Panel A: full sample period*											
Mean	0.001	0.001	0.001	0.001	0.001	0.001	0.001	0.001	0.001	0.001	0.001
Standard Deviation	0.023	0.022	0.022	0.029	0.027	0.025	0.024	0.025	0.027	0.028	0.080
Kurtosis	12.183	16.037	11.924	16.902	10.889	10.771	11.945	10.520	11.168	19.156	8.320
Skewness	− 0.031	0.136	− 0.057	− 0.031	− 0.246	0.206	0.095	0.059	− 0.201	0.388	0.768
Jarque–Bera	17,773.026	35,837.063	16,787.883	40,733.379	13,165.931	12,762.474	16,869.902	11,922.105	14,093.102	55,137.968	6462.790
*p-*value	0.000	0.000	0.000	0.000	0.000	0.000	0.000	0.000	0.000	0.000	0.000
ADF	− 67.162	− 65.570	− 70.149	− 68.661	− 69.408	− 68.698	− 69.851	− 69.248	− 67.750	–67.431	− 81.120
*p-*value	0.000	0.000	0.000	0.000	0.000	0.000	0.000	0.000	0.000	0.000	0.000
Observations	5058	5058	5058	5058	5058	5058	5058	5058	5058	5058	5058
*Panel B: GFC sample period*											
Mean	− 0.003	− 0.004	− 0.002	− 0.005	− 0.004	− 0.004	− 0.002	− 0.003	− 0.004	− 0.006	0.003
Standard Deviation	0.038	0.043	0.029	0.049	0.036	0.038	0.031	0.040	0.038	0.049	0.089
Kurtosis	7.792	6.691	4.721	11.104	4.754	7.468	3.453	4.586	3.621	3.579	1.558
Skewness	0.611	0.715	− 0.040	− 0.179	− 0.064	0.196	0.207	0.531	− 0.125	0.286	0.140
Jarque–Bera	356.731	228.464	43.286	959.608	45.124	293.398	5.492	53.111	6.522	9.665	31.468
*p-*value	0.000	0.000	0.000	0.000	0.003	0.000	0.000	0.000	0.000	0.000	0.000
ADF	− 13.465	− 13.329	− 16.272	− 14.691	− 15.067	− 16.279	− 14.939	− 15.333	− 13.398	− 15.021	− 18.508
*p-*value	0.000	0.000	0.000	0.000	0.000	0.000	0.000	0.000	0.000	0.000	0.000
Observations	350	350	350	350	350	350	350	350	350	350	350
*Panel C: COVID-19 sample Period*											
Mean	− 0.001	0.000	0.000	0.000	− 0.001	0.001	0.000	0.000	− 0.001	0.000	0.001
Standard Deviation	0.029	0.024	0.033	0.035	0.031	0.032	0.032	0.031	0.038	0.045	0.103
Kurtosis	7.783	6.596	5.689	7.435	6.166	4.513	4.976	7.915	5.355	9.673	4.006
Skewness	− 0.792	− 0.476	− 0.041	− 0.388	− 0.725	0.058	− 0.233	− 0.995	− 0.604	− 0.044	1.010
Jarque–Bera	370.256	201.860	105.538	295.679	176.875	33.561	60.135	410.161	102.175	649.537	74.266
*p-v*alue	0.000	0.000	0.000	0.000	0.000	0.000	0.000	0.000	0.000	0.000	0.000
ADF	− 13.409	− 14.155	− 14.520	− 12.740	− 14.556	− 14.613	− 14.374	− 14.415	− 15.926	− 11.984	− 18.653
*p-v*alue	0.000	0.000	0.000	0.000	0.000	0.000	0.000	0.000	0.000	0.000	0.000
Observations	350	350	350	350	350	350	350	350	350	350	350

This table reports the descriptive statistics of the full sample and the GFC/COVID-19 sub-samples of the study including mean, standard deviation, kurtosis, skewness, the Jarque–Bera normality test and Augmented Dickey–Fuller (ADF) unit-root test *p*-value. The variables are (with DataStream code in parentheses) the daily return of CREDIT SUISSE GROUP (S:CSGN), UBS(S:UBSG), BANCO SANTANDER (H:INGA), ING GROUP (H:INGA), UNICREDIT (I:UCG), DEUTSCHE BANK (D:DBK), BNP PARIBAS (F:BNP), CREDIT AGRICOLE (F:CRDA), SOCIETE GENERALE (F:SGE), NATIXIS (F:KN) and the CBOE Volatility Index VIX (CBOEVIX). The full sample period in Panel A spans the period from January 1, 2002, to May 21, 2021, the GFC period in Panel B is from August 1, 2007, to December 2, 2008, and the COVID-19 sample in Panel C is from January 1, 2020, to May 5, 2021

Table 1 reports key summary statistics. In the full sample, all returns (banks and VIX) display substantial kurtosis and the Jarque–Bera test rejects the null hypothesis of normality at the 1% level for all return series. This basic result is also confirmed in the two crisis samples. Moreover, all banks show negative skewness in the COVID-19 sample but not in the GFC sample. Negative skewness indicates a higher probability of extreme negative returns arising from the stock price collapse due to COVID-19. Note that stock markets in most developed countries fell more than 30% within the first few weeks^5^ of the COVID-19 pandemic in early 2020. Therefore, negative skewness is a characteristic feature of the COVID-19 sample. This difference is also indicated by the skewness of the VIX, which is 1.010 (COVID-19) compared with 0.140 (GFC). VIX’s skewness demonstrates that during COVID-19 extreme market panic (VIX jumps) was more pronounced than extreme market recovery (VIX drops).

**Fig. 1:**
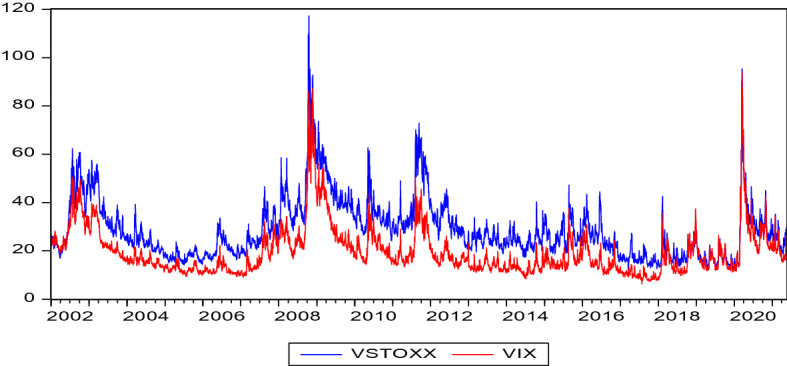
The figure plots daily levels of implied stock market volatility. VIX refers to the CBOE Volatility Index and VSTOXX is the European complement to the VIX measuring implied volatility of the Euro STOXX 50. The pairwise Pearson correlation is 0.90. The sample period is from January 2, 2002 to May 21, 2021

Figure 1 plots the daily evolution of the VIX and VSTOXX indices. Both implied volatility indices are highly correlated for our sample period, which is consistent with recent studies (e.g. Akyildirim et al., 2020; Clements et al., 2019). Also, the VIX is less volatile than the VSTOXX: with this feature, the VIX could be more appropriate for forecasting purposes. These findings underpin the preference in this study to use the VIX instead of the VSTOXX in the later analysis. In addition, Fig. 1 displays large levels of implied volatility during the GFC as well COVID-19. This highlights that jumps in implied volatility are a characteristic feature of extreme market crises. The plot also shows that the increase in implied volatility was faster during COVID-19 than during the GFC. This could be interpreted as the immediate impact of COVID-19 being more severe than the GFC, with the rapid increase in the implied volatility index reflecting market uncertainty about the impact of the pandemic on stock valuations.

## Results

This section includes the results from the empirical investigation of the linkage between the VIX and the sample banks as discussed in the previous sections. The results are presented in two categories: the baseline DCC-GARCH model with subsequent correlation analysis; and the secondary quantile regression analysis of asymmetric response of bank returns to changes in implied volatility during GFC and COVID-19.

### DCC-GARCH model results

Table 2 presents baseline results of the DCC-GARCH model in the full sample. The table reports estimated coefficients from our bivariate DCC-AR(4)-GARCH(1,3) model specification and associated *p-values*. Analyzed is the pairwise relation between daily VIX returns and returns of a set of European GSIBs for the sample period from January 1, 2002, to May 21, 2021. The Panel A of Table 2 contains the outcomes of the univariate conditional mean equation (see Eq. (3)) and Panel B the conditional variance equation (see Eq. (4)). Panel C then reports *Q*(*k*) and *Q*^2^(*k*), which provide results of the Ljung-Box test for serial correlation up to order *k,* applied to the standardized residuals and squared standardized residuals, individually. BIC is the Bayesian Information Criterion.

**Table 2:** DCC-AR(4)-GARCH(1,3) Model Results

	Credit suisse group	Ubs	Banco santander	Ing group	Unicredit
*Panel A: Mean Equation*					
*μ*	0.001**	0.001***	0.001***	0.001***	0.001**
*p*-value	0.015	0.006	0.003	0.004	0.013
$\rho_{1}$	−0.006	−0.031**	−0.060***	−0.033*	−0.036**
*p*-value	0.644	0.040	0.001	0.065	0.014
$\rho_{2}$	−0.021	−0.016	−0.020	−0.020	0.007
*p-v*alue	0.139	0.240	0.242	0.191	0.614
$\rho_{3}$	−0.030**	−0.015	−0.021	−0.033**	−0.025*
*p-*value	0.028	0.293	0.247	0.041	0.078
$\rho_{4}$	0.005	−0.003	0.003	0.001	0.007
*p-*value	0.717	0.777	0.816	0.989	0.579
$\lambda_{1}$	−0.033***	−0.029***	−0.026***	−0.032***	−0.022***
*p*-value	0.000	0.000	0.000	0.000	0.000
$\lambda_{2}$	−0.006**	−0.006**	−0.008**	−0.010***	−0.003
*p*-value	0.036	0.026	0.027	0.004	0.364
$\lambda_{3}$	−0.009***	−0.007**	−0.008**	−0.006*	−0.002
*p*-value	0.004	0.012	0.022	0.071	0.536
$\lambda_{4}$	0.003	0.003	−0.001	0.001	0.005
*p*-value	0.240	0.165	0.629	0.797	0.115
*Panel B: Variance Equation*					
$w_{0}$	0.000***	0.000***	0.000***	0.000***	0.000**
*p*-value	0.003	0.000	0.009	0.003	0.012
$w_{1}$	0.110***	0.106***	0.132***	0.145***	0.118***
*p*-value	0.000	0.000	0.005	0.000	0.000
$w_{2}$	0.890***	0.297***	0.698**	0.468**	0.702***
*p*-value	0.000	0.007	0.026	0.017	0.000
$w_{3}$	−0.405**	0.683***	0.139	0.339**	−0.156
*p*-value	0.049	0.000	0.492	0.014	0.673
$w_{4}$	0.406***	−0.092	0.027	0.044	0.343
*p*-value	0.000	0.474	0.837	0.696	0.133
*Panel C: DCC Equation*					
*a*	0.010***	0.008***	0.014***	0.015	0.010***
*p*-value	0.001	0.005	0.002	0.320	0.000
*b*	0.977***	0.976***	0.069***	0.968***	0.982***
*p*-value	0.000	0.000	0.000	0.000	0.000
*Panel D: Diagnostics*					
Log-likelihood	19,178.580	20,348.150	19,508.44	19,005.880	18,993.280
BIC	−38,084.280	−40,423.410	−38,752.510	−37,747.390	−37,713.670
*Q* (5)	5.011	6.988	5.084	13.762**	6.963
*Q *(10)	11.218	10.768	6.461	16.574*	9.561
$Q^{2} \left( 5 \right)$	11.107*	7.506	8.337	8.009	14.772**
$Q^{2} \left( {10} \right)$	13.601	10.312	11.977	13.341	16.588*

	Deutsche bank	Bnp paribas	Credit agricole	Societe generale	Natixis
*Panel A: Mean Equation*					
*μ*	0.001*	0.001***	0.001**	0.001***	0.001*
*p*-value	0.061	0.007	0.020	0.010	0.059
$\rho_{1}$	−0.002	−0.036**	−0.029	−0.028*	0.001
*p*-value	0.851	0.016	0.179	0.058	0.929
$\rho_{2}$	0.007	−0.018	−0.010	−0.020	−0.012
*p-v*alue	0.625	0.197	0.529	0.159	0.381
$\rho_{3}$	−0.025*	−0.036**	−0.028*	−0.013	−0.024*
*p-*value	0.093	0.011	0.094	0.324	0.084
$\rho_{4}$	0.014	−0.016	−0.025	0.005	−0.002
*p-*value	0.321	0.259	0.157	0.660	0.840
$\lambda_{1}$	−0.018***	−0.030***	−0.028***	−0.022***	−0.033***
*p*-value	0.000	0.000	0.000	0.000	0.000
$\lambda_{2}$	−0.002	−0.003	−0.005	−0.001	−0.010***
*p*-value	0.485	0.231	0.240	0.873	0.001
$\lambda_{3}$	−0.003	−0.005*	−0.002	−0.001	−0.008***
*p*-value	0.378	0.051	0.446	0.614	0.006
$\lambda_{4}$	0.006*	0.001	−0.001	0.005*	0.003
*p*-value	0.098	0.684	0.713	0.067	0.182
*Panel B: Variance Equation*					
$w_{0}$	0.000	0.000***	0.000	0.000***	0.000***
*p*-value	0.280	0.001	0.302	0.004	0.001
$w_{1}$	0.053	0.090***	0.112	0.160***	0.100***
*p*-value	0.235	0.000	0.326	0.000	0.000
$w_{2}$	1.324***	0.733***	0.541	0.544*	0.376***
*p*-value	0.002	0.005	0.595	0.053	0.008
$w_{3}$	−0.773*	0.166	0.183	0.131	0.468***
*p*-value	0.056	0.508	0.281	0.690	0.001
$w_{4}$	0.394	0.006	0.154	0.173	0.045
*p*-value	0.575	0.949	0.846	0.650	0.653
*Panel C: DCC Equation*					
*a*	0.008***	0.010***	0.012*	0.008***	0.010***
*p*-value	0.000	0.008	0.092	0.000	0.000
*b*	0.987***	0.979***	0.972***	0.981***	0.975***
*p*-value	0.000	0.000	0.000	0.000	0.000
*Panel D: Diagnostics*					
Log-likelihood	19,454.210	19,680.520	18,815.340	19,351.020	19,688.32
BIC	-38,635.530	−39,088.14	−37,366.32	−38,638.050	−39,103.76
*Q* (5)	3.047	4.319	7.087	3.847	2.123
*Q *(10)	10.785	9.193	8.156	5.516	8.304
*Q*^*2*^(5)	5.365	10.58*	5.137	2.107	4.892
$Q^{2} \left( {10} \right)$	13.250	15.086	6.453	4.681	7.140

The table reports estimated coefficients from the bivariate DCC-AR(4)-GARCH(1,3) model and associated *p*-values. Analyzed is the pairwise relation between daily VIX returns and returns of a set of European GSIBs (with DataStream code in parentheses) including CREDIT SUISSE GROUP (S:CSGN), UBS(S:UBSG), BANCO SANTANDER (H:INGA), ING GROUP (H:INGA), UNICREDIT (I:UCG), DEUTSCHE BANK (D:DBK), BNP PARIBAS (F:BNP), CREDIT AGRICOLE (F:CRDA), SOCIETE GENERALE (F:SGE), NATIXIS (F:KN) for the sample period of January 1, 2002 to May 21, 2021. The first part of the result contains the outcome of the univariate conditional mean equation (see Eq. (3)) and conditional variance equation (see Eq. (4)). *Q*(*k*) and *Q*^2^(*k*) characterize the Ljung-Box test for serial correlation up to order *k* applied to standardized residuals and squared standardized residuals, individually. BIC is the Bayesian Information Criterion. The 1, 5 and 10% significance levels are denoted by ***, ** and *, respectively

Following the results presented in Table 2, both DCC parameters $a$ and $b$, in most cases, are highly significant at the 1% level. As the correlation among our variables is time-varying, this finding is in line with previous statistical evidence of time-varying correlation (e.g. Akkoc & Civcir, 2019; Shiferaw, 2019). Thus, there is a strong time-varying relationship between the VIX and our sample of bank returns. We can also observe a highly significant parameter $\lambda_{1}$ as a clear indication of Granger causality. This relationship shows that a previous day's jump in VIX has a negative impact on the stock returns of the sample banks. This finding is also consistent with other recent studies and is consistent with the VIX being used to hedge asset price shocks (Ding et al., 2021).

In the diagnostics tests (Panel C), the BIC parameter shows a similar model fit for all pairs. The DCC-AR(4)-GARCH(1,3) model is specified appropriately by analyzing standardized and squared standardized residuals on serial correlation. The Ljung-Box test is used for low (i.e., lag 5) and high (i.e., lag 10) orders of serial correlation. We conclude that our model is appropriate for this data.

**Fig. 2:**
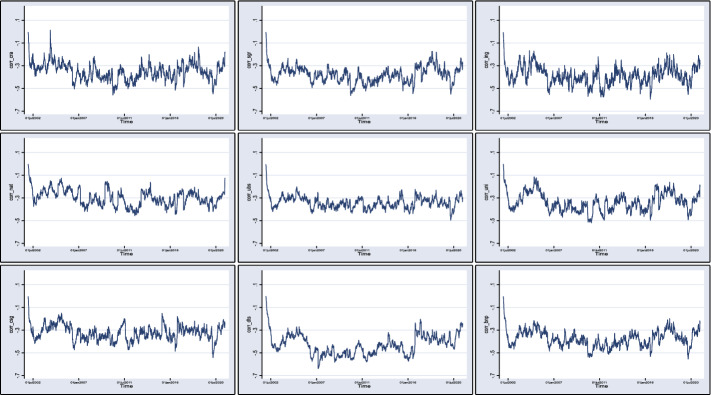
Full Sample Correlation. This figure plots the time-varying conditional correlations (see Eq. (7)) arising from the bivariate DCC-AR(4)-GARCH(1,3) model. Plotted are the pairwise correlations between VIX and the following banks: CREDIT SUISSE GROUP (corr_csg), UBS(corr_ubs), ING GROUP (corr_ing), UNICREDIT (corr_uni), DEUTSCHE BANK (corr_dts), BNP PARIBAS (corr_bnp), CREDIT AGRICOLE (corr_cra), SOCIETE GENERALE (corr_sgr) and NATIXIS (corr_nat) for the sample period of January 1, 2002, to May 21, 2021. We have omitted BANCO SANTANDER as it is visually similar with BNP PARIBAS

Next in Fig. 2, we plot the time-varying conditional correlations (see Eq. (7)) that arise from our previous bivariate DCC-AR(4)-GARCH(1,3) model for the sample period from January 1, 2002, to May 21, 2021. BANCO SANTANDER is omitted as it is visually similar to BNP PARIBAS. The figure highlights the time-varying nature of the correlations that fluctuate between -0.6 to 0.0, with CRA and DTS displaying the most fluctuation, while UBS and UNI display the least.

**Fig. 3:**
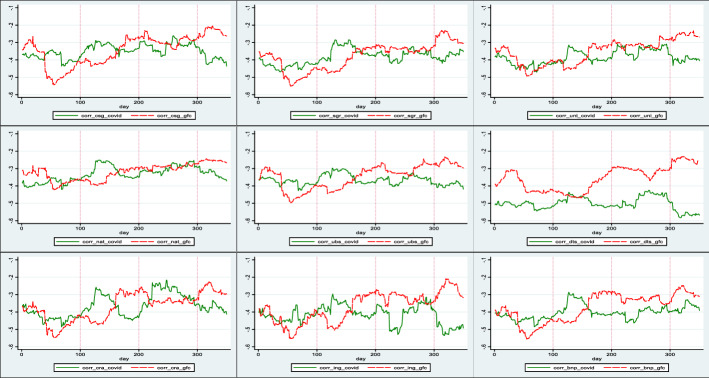
Sample Correlation for the first 350 crisis days: GFC vs COVID-19. This figure plots the time-varying conditional correlations (see Eq. (7)) arising from the bivariate DCC-AR(4)-GARCH(1,3) model for the first 350 days of GFC and COVID-19. Plotted are pairwise correlations between VIX and the following banks: CREDIT SUISSE GROUP (corr_csg), UBS(corr_ubs), ING GROUP (corr_ing), UNICREDIT (corr_uni), DEUTSCHE BANK (corr_dts), BNP PARIBAS (corr_bnp), CREDIT AGRICOLE (corr_cra), SOCIETE GENERALE (corr_sgr) and NATIXIS (corr_nat). The GFC period has been stated as August 1, 2007, to December 2, 2008 and the COVID-19 sample is consists of daily data of January 1, 2020, to May 5, 2021.We have omitted BANCO SANTANDER as it is visually similar with BNP PARIBAS

To further investigate the impact of these correlations in the GFC and COVID-19 periods, in Fig. 3, the time-varying conditional correlations for the first 350 days of these crises, are plotted. This figure plots the time-varying conditional correlations (see Eq. (7)) arising from the bivariate DCC-AR(4)-GARCH(1,3) model. The GFC period has been stated as August 1, 2007, to December 2, 2008, and the COVID-19 sample consists of daily data from January 1, 2020, to May 5, 2021. We again omit BANCO SANTANDER as it is visually similar to the correlation of BNP PARIBAS.

First, we can observe three clearly distinguishable patterns in these charts. NAT, UNI, and SGR follow a consistent pattern, where the GFC and COVID-19 line stays close to one another. The correlation value also rises in the last half of the chart. This may be compared with DTS where the correlation of COVID-19 lags the GFC. The other graphs from the remaining five banks follow the same market movement as the first pattern, but with greater fluctuation. Interestingly, during COVID-19 the correlation was above the GFC correlation in the first half of the plot, but below in the second half. We can conclude that as COVID-19 progressed, the impact of VIX had a greater influence on the sample banks compared to the GFC.

### Asymmetric impact of relative VIX changes on bank returns

#### GFC vs COVID-19

The asymmetric impact of relative VIX changes (i.e. returns) on bank returns using Eq. (8), is investigated using quantile regression. Tables 3 and 4 report these results for the regressions at the 25, 50, and 75% quantile, for the GFC and COVID-19 sample.

**Table 3:** Impact of relative VIX changes on bank returns in the GFC sample

	Credit suisse group	Ubs	Banco santander	Ing group	Unicredit	Deutsche bank	Bnp paribas	Credit agricole	Societe generale	Natixis
*Panel A: 25% Quantile*										
$VIX\_U$	− 0.136**	− 0.191***	− 0.211***	− 0.300***	− 0.220***	− 0.293***	− 0.120***	− 0.221***	− 0.208***	− 0.158**
*p*-value	0.045	0.000	0.000	0.002	0.000	0.000	0.000	0.001	0.001	0.046
$VIX\_D$	− 0.088***	− 0.130**	− 0.098***	− 0.082*	− 0.049	− 0.094***	− 0.131**	− 0.053	0.017	− 0.171***
*p*-value	0.001	0.041	0.004	0.068	0.364	0.001	0.024	0.465	0.744	0.001
$Constant$	− 0.015***	− 0.022***	− 0.010***	− 0.013***	− 0.012***	− 0.011***	− 0.016***	− 0.017***	− 0.014***	− 0.028***
*p*-value	0.000	0.000	0.000	0.000	0.001	0.000	0.000	0.000	0.000	0.000
*Panel B: 50% Quantile*										
$VIX\_U$	− 0.086***	− 0.140***	− 0.099**	− 0.132***	− 0.151***	− 0.165***	− 0.131***	− 0.113***	− 0.159***	− 0.122**
*p*-value	0.000	0.002	0.018	0.002	0.001	0.000	0.000	0.001	0.000	0.031
$VIX\_D$	− 0.066**	− 0.090*	− 0.091**	− 0.118**	− 0.108***	− 0.139***	− 0.082***	− 0.108***	− 0.031	− 0.135***
*p*-value	0.011	0.091	0.043	0.034	0.006	0.007	0.001	0.008	0.442	0.000
$Constant$	− 0.004*	− 0.003	− 0.002	− 0.001	− 0.001	− 0.002	− 0.003*	− 0.004*	0.000***	− 0.005
*p*-value	0.088	0.206	0.308	0.731	0.589	0.230	0.057	0.087	0.834	0.148
*Panel C: 75% Quantile*										
$VIX\_U$	− 0.085*	− 0.098***	− 0.048*	− 0.095**	− 0.098***	− 0.116***	− 0.100***	− 0.124***	− 0.151***	− 0.140***
*p*-value	0.068	0.001	0.055	0.030	0.010	0.000	0.001	0.000	0.000	0.001
$VIX\_D$	− 0.148	− 0.228***	− 0.181***	− 0.272**	− 0.184***	− 0.219***	− 0.163***	− 0.144	− 0.040	− 0.178***
*p*-value	0.214	0.001	0.004	0.042	0.003	0.000	0.008	0.129	0.566	0.004
$Constant$	0.008**	0.010***	0.006*	0.009**	0.009***	0.007***	0.011***	0.013***	0.017***	0.016***
*p*-value	0.044	0.008	0.061	0.011	0.002	0.000	0.000	0.000	0.000	0.000

The table reports estimated coefficients and associated *p*-values from a quantile regression of daily bank returns on relative changes in VIX. *VIX*_*U* and *VIX*_*D* denote relative positive (negative) changes in VIX. The quantile regression is performed for the 0.25, 0.50 (median) and 0.75 quantile. All panels report results based on a GFC sample from August 1, 2007 to December 2, 2008. The 1, 5 and 10% significance levels are denoted by ***, ** and *, respectively

**Table 4:** Impact of relative VIX changes on bank returns in the COVID-19 sample

	Credit suisse group	Ubs	Banco santander	Ing group	Unicredit	Deutsche bank	Bnp paribas	Credit agricole	Societe generale	Natixis
*Panel A: 25% Quantile*										
$VIX\_U$	− 0.157***	− 0.149***	− 0.191***	− 0.180***	− 0.176**	− 0.174***	− 0.237***	− 0.215***	− 0.191**	− 0.179***
*p*-value	0.001	0.000	0.000	0.005	0.016	0.004	0.001	0.000	0.047	0.005
$VIX\_D$	0.011	− 0.021	0.006	0.028	0.011	− 0.024	− 0.001	0.006	− 0.050	0.043
*p*-value	0.724	0.476	0.846	0.389	0.823	0.521	0.979	0.904	0.452	0.207
$Constant$	− 0.007***	− 0.007***	− 0.012***	− 0.008**	− 0.010***	− 0.010***	− 0.008***	− 0.009***	− 0.013***	− 0.005***
*p*-value	0.004	0.003	0.000	0.045	0.000	0.000	0.000	0.000	0.001	0.003
*Panel B: 50% Quantile*										
$VIX\_U$	− 0.108***	− 0.067*	− 0.116***	− 0.109***	− 0.112***	− 0.100***	− 0.104***	− 0.113***	− 0.123**	− 0.116***
*p*-value	0.007	0.088	0.001	0.000	0.002	0.001	0.006	0.000	0.016	0.004
$VIX\_D$	− 0.017	− 0.044	− 0.028	− 0.009	− 0.048*	− 0.045**	− 0.062***	− 0.054*	− 0.057	− 0.001
*p*-value	0.592	0.172	0.458	0.679	0.099	0.042	0.004	0.079	0.213	0.980
$Constant$	0.001	0.001	0.000	0.002	0.000	0.001	0.001	0.002	0.001	0.003
*p*-value	0.613	0.728	0.779	0.160	0.812	0.385	0.667	0.556	0.627	0.151
*Panel C: 75% Quantile*										
$VIX\_U$	− 0.051	− 0.041	− 0.127***	− 0.130***	− 0.083**	− 0.099***	− 0.114**	− 0.104***	− 0.145***	− 0.078
*p*-value	0.114	0.172	0.001	0.001	0.011	0.000	0.011	0.000	0.000	0.129
$VIX\_D$	− 0.079**	− 0.032	− 0.078	− 0.039	− 0.117**	− 0.051	− 0.041	− 0.069**	− 0.107*	− 0.080
*p*-value	0.014	0.214	0.253	0.400	0.026	0.482	0.350	0.038	0.055	0.276
$Constant$	0.010***	0.011***	0.019***	0.019***	0.013***	0.018***	0.017***	0.015***	0.018***	0.014***
*p*-value	0.002	0.000	0.000	0.000	0.000	0.000	0.000	0.000	0.000	0.000

The table reports estimated coefficients and associated *p*-values from a quantile regression of daily bank returns on relative changes in VIX. *VIX*_*U* and *VIX*_*D* denote relative positive (negative) changes in VIX. The quantile regression is performed for the 0.25, 0.50 (median) and 0.75 quantile. All panels report results based on a COVID-19 sample from January 1, 2020 to May 5, 2021. The 1, 5 and 10% significance levels are denoted by ***, ** and *, respectively

Following the findings in the previous section, there is clear evidence of a strong relationship between $VIX_{U,t}$ and bank returns at the time of the GFC and COVID-19 in the 25% quantile. Jumps in VIX have a significant negative impact on bank returns. This is documented in all quantiles, with the most pronounced effect visible in the 25% quantile. In addition, during COVID-19, all banks show a significant effect for $VIX_{U,t}$, but there is no significance for $VIX_{D,t}$ in the 25% quantile. This finding clearly demonstrates the asymmetric impact of changes in VIX on (negative) bank returns during COVID-19. However, during the GFC there is also asymmetry, although it is less pronounced: $VIX_{D,t}$ is significant at least at the 5% level for only half of the banks. Only for CSG and NAT are negative changes in VIX more significant than positive ones. These findings are related to the previous finding in Sect. 4, where the preliminary analysis shows a higher positive skewness of VIX in COVID-19 (see also the findings of Shehzad et al. (2020)). However, these previous results do not establish if the positive skewness in VIX implies an asymmetric response to bank returns.

The constant in Panel A of Tables 3 and 4 shows significant negative values, which demonstrate that this quantile is related primarily to losses in prices. Given this finding and the results of the quantile regression, we can conclude that changes in VIX in COVID-19 had a more severe impact on bank returns, with jumps in VIX significantly related to bank returns, while drops in VIX are not. A possible economic explanation could be due to the sudden -and over a very short time period- decline in stock markets due to COVID-19, with the impact of crisis not restricted to a few industries, as occurred during the GFC. As a result, the capital market impact of COVID-19 was significant and correlates with the relation between jumps in VIX and bank returns.^6^

#### Robustness check: swine Flu and Zika virus

Following the previous analysis, the asymmetric impact of the VIX on our sample of bank returns is compared with two other severe health stress scenarios: The Swine Flu and Zika virus epidemics. Using the model in Eq. (8), Tables 5 and 6 report the quantile regression results.

**Table 5:** Impact of relative VIX changes on bank returns in the Swine Flu (H1N1) sample

	Credit suisse group	Ubs	Banco santander	Ing group	Unicredit	Deutsche bank	Bnp paribas	Credit agricole	Societe generale	Natixis
*Panel A: 25% Quantile*										
$VIX\_U$	− 0.186***	− 0.208***	− 0.276***	− 0.371***	− 0.264***	− 0.268***	− 0.322***	− 0.317***	− 0.290***	− 0.338***
*p*-value	0.000	0.000	0.000	0.000	0.000	0.000	0.000	0.000	0.000	0.000
$VIX\_D$	− 0.089**	− 0.133***	− 0.110***	− 0.134*	− 0.123***	− 0.116*	− 0.120**	− 0.071*	− 0.147***	− 0.020
*p*-value	0.011	0.001	0.008	0.063	0.001	0.062	0.032	0.058	0.004	0.708
$Constant$	− 0.010***	− 0.010***	− 0.007**	− 0.010***	− 0.011***	− 0.008***	− 0.007***	− 0.010***	− 0.012***	− 0.009***
*p*-value	0.000	0.000	0.001	0.000	0.000	0.007	0.000	0.000	0.000	0.000
*Panel B: 50% Quantile*										
$VIX\_U$	− 0.196***	− 0.205***	− 0.273***	− 0.322***	− 0.261***	− 0.193***	− 0.271***	− 0.289***	− 0.287***	− 0.280***
*p*-value	0.000	0.000	0.000	0.000	0.000	0.000	0.000	0.000	0.000	0.000
$VIX\_D$	− 0.077***	− 0.127***	− 0.124*	− 0.205***	− 0.152**	− 0.143***	− 0.196***	− 0.121**	− 0.173***	− 0.143*
*p*-value	0.001	0.000	0.082	0.000	0.016	0.000	0.000	0.018	0.001	0.054
$Constant$	0.002	0.002	0.003	0.002	0.001	0.001	0.000	0.003	0.002	0.003
*p*-value	0.313	0.316	0.119	0.194	0.718	0.440	0.764	0.216	0.255	0.408
*Panel C: 75% Quantile*										
$VIX\_U$	− 0.174***	− 0.202***	− 0.174***	− 0.331***	− 0.235***	− 0.172***	− 0.248***	− 0.261***	− 0.278***	− 0.229***
*p*-value	0.000	0.000	0.001	0.000	0.000	0.000	0.000	0.000	0.000	0.000
$VIX\_D$	− 0.095**	− 0.123**	− 0.288***	− 0.284***	− 0.187***	− 0.182***	− 0.203**	− 0.134***	− 0.183***	− 0.143**
*p*-value	0.022	0.011	0.000	0.000	0.000	0.001	0.014	0.003	0.000	0.037
$Constant$	0.013***	0.016***	0.010***	0.018***	0.015***	0.012***	0.014***	0.014***	0.015***	0.017***
*p*-value	0.000	0.000	0.000	0.000	0.000	0.000	0.000	0.000	0.000	0.000

The table reports estimated coefficients and associated *p*-values from a quantile regression of daily bank returns on relative changes in VIX. *VIX*_*U* and *VIX*_*D* denote relative positive (negative) changes in VIX. The quantile regression is performed for the 0.25, 0.50 (median) and 0.75 quantile. All panels report results based on a Swine Flu (H1N1) sample from April 25, 2009 to August 27, 2010. The 1, 5 and 10% significance levels are denoted by ***, ** and *, respectively

**Table 6:** Impact of relative VIX changes on bank returns in the Zika virus sample

	Credit suisse group	Ubs	Banco santander	Ing group	Unicredit	Deutsche bank	Bnp paribas	Credit agricole	Societe generale	Natixis
*Panel A: 25% Quantile*										
$VIX\_U$	− 0.200**	− 0.14*	− 0.224**	− 0.217***	− 0.186	− 0.251**	− 0.170***	− 0.196***	− 0.244***	− 0.201**
*p*-value	0.032	0.065	0.045	0.002	0.253	0.028	0.007	0.007	0.008	0.043
$VIX\_D$	− 0.002	− 0.004	− 0.009	− 0.007	− 0.007	− 0.007	− 0.009	− 0.009	− 0.007	− 0.009
*p*-value	0.988	0.954	0.935	0.944	0.957	0.959	0.938	0.930	0.957	0.938
$Constant$	− 0.009**	− 0.005*	− 0.007*	− 0.004	− 0.017**	− 0.010*	− 0.006**	− 0.005	− 0.005	− 0.008*
*p*-value	0.044	0.065	0.073	0.172	0.017	0.086	0.04	0.100	0.207	0.081
*Panel B: 50% Quantile*										
$VIX\_U$	− 0.090	− 0.087	− 0.127	− 0.093*	− 0.161	− 0.154	− 0.103	− 0.108	− 0.094	− 0.088
*p*-value	0.187	0.260	0.304	0.097	0.273	0.212	0.218	0.208	0.325	0.258
$VIX\_D$	− 0.124	− 0.057	− 0.120	− 0.084	− 0.028	− 0.073	− 0.120	− 0.085	− 0.128	− 0.097
*p*-value	0.309	0.488	0.272	0.289	0.861	0.627	0.240	0.365	0.295	0.471
$Constant$	− 0.001	0.000	0.002	0.000	0.001	0.000	0.000	0.000	0.000	0.000
*p*-value	0.776	1.000	0.682	1.000	0.892	0.990	1.000	0.849	1.000	1.000
*Panel C: 75% Quantile*										
$VIX\_U$	− 0.013	− 0.008	− 0.020	− 0.014	− 0.016	− 0.018	− 0.023	− 0.015	− 0.017	0.005
*p*-value	0.790	0.914	0.854	0.718	0.913	0.875	0.749	0.808	0.835	0.937
$VIX\_D$	− 0.191	− 0.165*	− 0.238*	− 0.247***	− 0.227	− 0.189	− 0.145	− 0.205*	− 0.229**	− 0.213
*p*-value	0.165	0.069	0.051	0.001	0.247	0.200	0.148	0.051	0.024	0.100
$Constant$	0.008**	0.004	0.007	0.003*	0.010*	0.009	0.008**	0.004*	0.007**	0.006
*p*-value	0.042	0.245	0.143	0.078	0.091	0.105	0.024	0.095	0.025	0.202

The table reports estimated coefficients and associated *p*-values from a quantile regression of daily bank returns on relative changes in VIX. *VIX*_*U* and *VIX*_*D* denote relative positive (negative) changes in VIX. The quantile regression is performed for the 0.25, 0.50 (median) and 0.75 quantile. All panels report results based on a Zika virus sample from November 17, 2015 to March 20, 2017. The 1, 5 and 10% significance levels are denoted by ***, ** and *, respectively

These results show a strong negative relationship in the 25% quantile, following the GFC and COVID-19 results, for both Swine Flu and Zika virus sample. However, the Zika virus sample does not show any impact on $VIX_{D,t}$ in the lower quartile and almost no impact in the other quartiles. We observe a strong negative relationship in the 25% quantile for $VIX_{U,t}$. These results are consistent for Swine Flu, where $VIX_{U,t}$ is more influential than its counterpart $VIX_{D,t}$, and clearly demonstrate the asymmetric impact of changes in VIX on bank returns during both the Swine Flu and Zika virus period.

## Conclusion

In this study, we investigate the volatility transmission between the VIX and European GSIBs with particular attention given to behavior over the GFC and COVID-19 periods. The findings show a significant negative time-varying correlation among the sample banks. During COVID-19 there was a higher negative correlation and an asymmetric effect of changes in VIX on extreme negative bank returns, in the lower quartile. This effect is more pronounced during COVID-19 than during the GFC. These results are consistent across additional analysis conducted during the periods of the Swine Flu and Zika virus epidemics.

Our results help explain contagion during periods of unusually high market stress and extreme movement in asset prices. The asymmetric nature of VIX impacts also has important implications for future research in modeling market behavior. Practitioners and regulators can use our findings to help explain the differences, as well as the similarities, between financial crises, and thereby develop better explanations of how market risk interacts with asset prices. Overall, this area of research offers wide social and economic benefits in the form of better understanding of the drivers of financial instability and the ability to manage risk.

While the GFC was a financial crisis, with a longer-term economic impact (and recovery) on financial markets, the impact of the COVID-19 pandemic on market prices and the VIX was more immediate. We show in this study that these impacts immediately affected bank stock prices, likely due to concerns over the possible deterioration in bank asset values. One likely reason for the shorter duration of market stress that arose from COVID-19, was the immediate monetary response by central banks to maintain market liquidity and support bank funding, as well as the fiscal support provided by government. This may be compared with the response during the GFC, where there was a long lead up to the implementation of the first Quantitative Easing (QE) and fiscal measures. However, QE measures are not without consequence, since they may trigger higher levels of future inflation and long-term deterioration in bank asset values. These possible impacts could be investigated in future research.

## Footnotes

1 The Bank for International Settlements (2021) Table B1-S (Summary of consolidated statistics, by nationality of reporting bank) estimate reporting bank consolidated (both domestic and foreign) assets in March 2021 as US$99.7 trillion. This data does not report assets of banks in mainland China. Banks in the European Union had assets of US$35.8 trillion, while the United States had assets of US$18.84 trillion.

2 Over the past 20 years the two highest scores of the VIX were on November 21, 2008 during the GFC (with the VIX closing at 80.74) and on March 16, 2020, during the inset of COVID-19 (the VIX closed at 82.69; the highest level since its inception in 1990).

3 VSTOXX refers to the EURO STOXX 50, and is a stock index of Eurozone stocks and is dominated by stocks listed in France and Germany: https://www.stoxx.com/index-details?symbol=V2TX.

4 These banks are commonly regarded as sytemically important, see GSIB list of the Financial Stability Board: https://www.fsb.org/wp-content/uploads/P111120.pdf.

5 The STOXX Europe 600, the broadest measure of European stock markets declined nearly 32% from February 19, 2020 (430.54) to March 20, 2020 (293.04).

6 In our analysis, the COVID-19 sample period ends in May 2021, which covers the period of most severe market stress since the occurence of the pandemic. However, this does not mean that COVID-19 has already ended. It is likely that further market turmoil could occur due to unforeseen developments, such as COVID-19 mutations.
