# Correction to: Volatility impacts on the European banking sector: GFC and COVID-19

**DOI:** 10.1007/s10479-022-04639-x

**Published:** 2022-03-03

**Authors:** Jonathan A. Batten, Tonmoy Choudhury, Harald Kinateder, Niklas F. Wagner

**Affiliations:** 1 RMIT University College of Business, Melbourne, VIC Australia; 2 IRC of Finance and Digital Economy, King Fahd University of Petroleum & Minerals, Dhahran, Kingdom of Saudi Arabia; 3 School of Business, Economics and Information Systems, University of Passau, Passau, Germany

## Correction to: Annals of Operations Research 10.1007/s10479-022-04523-8

This erratum is published as several proof corrections were overlooked during typesetting.

Original article has been updated.

